# Supplementary material for: Resolving the full spectrum of human genome variation using Linked-Reads
Source: Genome Res. 2019 Apr;29(4):635–45. doi: 10.1101/gr.234443.118 (PMC6442396; doi:10.1101/gr.234443.118)
Supplement: Supplemental Material [file supp_gr.234443.118_Supplemental_longranger-2.2.zip › longranger-2.2.tar.bz2!/lr2.2-public/lib/stan/cmdstan/stan/doc/api/html/stepsize__adapter_8hpp_source.html]

Stan: src/stan/mcmc/stepsize\_adapter.hpp Source File


|  |  |
| --- | --- |
|  | Stan  2.5.0  probability, sampling & optimization |


- Main Page
- Related Pages
- Namespaces
- Classes
- Files

- File List
- File Members

All Classes Namespaces Files Functions Variables Typedefs Enumerations Enumerator Friends Macros Pages

- src
- stan
- mcmc

stepsize\_adapter.hpp

Go to the documentation of this file.

1 #ifndef STAN\_\_MCMC\_\_STEPSIZE\_\_ADAPTER\_\_BETA

2 #define STAN\_\_MCMC\_\_STEPSIZE\_\_ADAPTER\_\_BETA

3

4 #include <stan/mcmc/base\_adapter.hpp>

5 #include <stan/mcmc/stepsize\_adaptation.hpp>

6

7 namespace stan {

8

9  namespace mcmc {

10

11  class stepsize\_adapter: public base\_adapter {

12

13  public:

14

15  stepsize\_adapter() {};

16

17  stepsize\_adaptation& get\_stepsize\_adaptation() {

18  return stepsize\_adaptation\_;

19  }

20

21  protected:

22

23  stepsize\_adaptation stepsize\_adaptation\_;

24

25  };

26

27  } // mcmc

28

29 } // stan

30

31 #endif

stan::mcmc::stepsize\_adaptation

**Definition:** stepsize\_adaptation.hpp:11

stan::mcmc::base\_adapter

**Definition:** base\_adapter.hpp:8

stepsize\_adaptation.hpp

stan::mcmc::stepsize\_adapter::stepsize\_adapter

stepsize\_adapter()

**Definition:** stepsize\_adapter.hpp:15

stan::mcmc::stepsize\_adapter::get\_stepsize\_adaptation

stepsize\_adaptation & get\_stepsize\_adaptation()

**Definition:** stepsize\_adapter.hpp:17

stan::mcmc::stepsize\_adapter::stepsize\_adaptation\_

stepsize\_adaptation stepsize\_adaptation\_

**Definition:** stepsize\_adapter.hpp:23

stan::mcmc::stepsize\_adapter

**Definition:** stepsize\_adapter.hpp:11

base\_adapter.hpp


---

[ Stan Home Page ]

*© 2011–2014,
Stan Development Team.*
